# Supplementary material for: Compounding impact of severe weather events fuels marine heatwave in the coastal ocean
Source: Nat Commun. 2020 Sep 22;11:4623. doi: 10.1038/s41467-020-18339-2 (PMC7508827; doi:10.1038/s41467-020-18339-2)
Supplement: Supplementary file 1 — Supplementary Information [file 41467_2020_18339_MOESM1_ESM.pdf]

Supplementary Materials for

# Compounding impact of severe weather events fuels marine heatwave in the coastal ocean

Brian Dzwonkowski, Jeff Coogan, Severine Fournier, Grant Lockridge, Kyeong Park, and Tong Lee

Correspondence to: briandz@disl.org

**This PDF file includes:**

Supplementary Fig. 1

**Supplementary Fig. 1: Time series of water and air temperatures.**

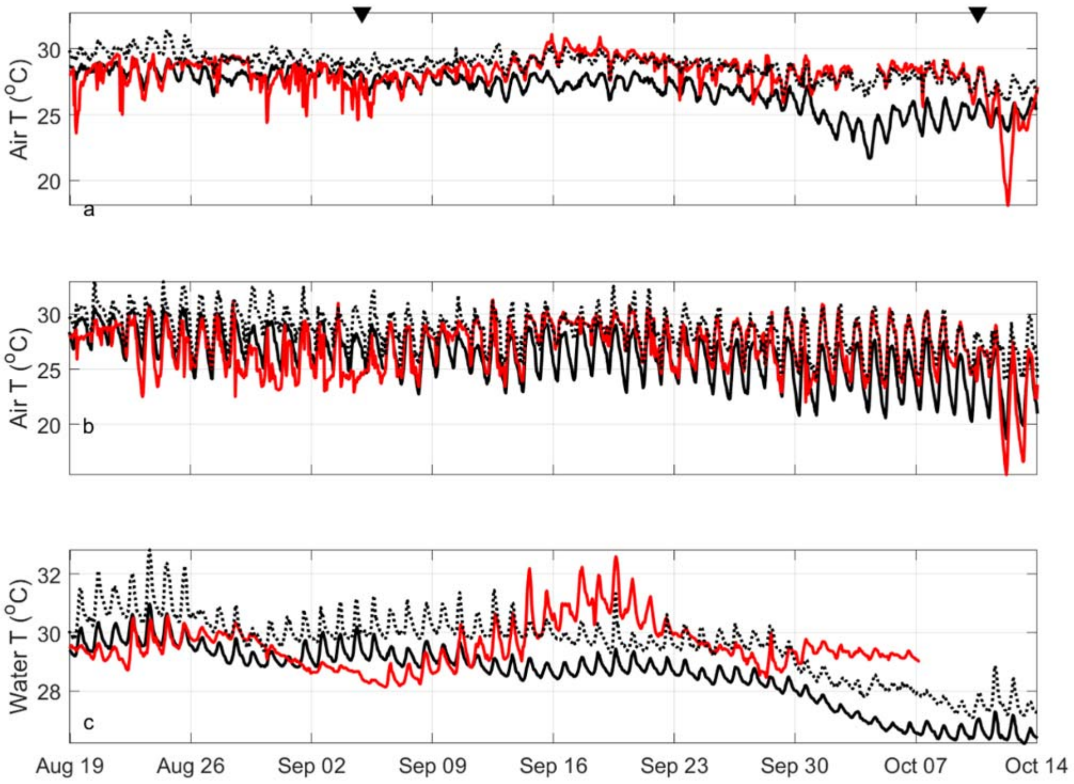


**Supplementary Fig. 1: Time series of water and air temperatures.** Time series during the fall of 2018 for (**a**) air temperature at ORB (red), (**b**) air temperature at PCB (red), and (**c**) SST at site CP (red), with the long-term mean (black line) and the 90$^{th}$ percentile threshold (dotted line) based on the available 10, 5-9, and 9 (based on data at ORB) years of data, respectively. In (**a**), the black triangles indicate landfall times of TS Gordon (9/4/2018) and Hurricane Michael (10/10/2018).
